# Arterial Thrombosis in Patients with Cancer

**DOI:** 10.3390/cancers16122238

**Published:** 2024-06-17

**Authors:** Yan Xu, Marc Carrier, Miriam Kimpton

**Affiliations:** Department of Medicine, The Ottawa Hospital Research Institute at University of Ottawa, Ottawa, ON K1H 8L6, Canada; yaxu@toh.ca (Y.X.); mkimpton@ohri.ca (M.K.)

**Keywords:** embolism and thrombosis, arteries, neoplasms

## Abstract

**Simple Summary:**

Patients with cancer are at high risk of blood clots in the arteries, leading to complications, such as stroke and heart attack. In this review, we discuss the risk factors associated with arterial blood clots in cancer patients, provide explanations on the cause of the arterial blood clots, and report on the current strategies for the prevention and treatment of arterial blood clots in cancer patients.

**Abstract:**

Patients with cancer are at increased risk of arterial thromboembolic disease due to the presence of risk factors common to both the development of cancer and arterial thrombosis, the cancer itself, and the treatments provided to treat cancer. We review here the epidemiology and pathophysiology of arterial thromboembolic disease in cancer, along with its prevention and treatment strategies. We also propose a generalized approach for the management of arterial thromboembolic disease in this patient population.

## 1. Introduction

### 1.1. Case 1

A 75-year-old woman with stage IIIB non-small lung cancer is referred for assessment of antithrombotic therapy in the context of a cerebellar infarct. The patient had non-resectable disease at diagnosis but had not yet started systemic therapy. Upon presentation to the ED with transient hemiparesis, dysphasia, and vertigo, a brain and neck computed tomogram with and without angiography queried the presence of cerebellar hypodensity, subsequently confirmed on magnetic resonance imaging to be early subacute infarcts in the left cerebellar hemisphere, right cerebellar hemisphere, and the right pons. No large vessel occlusion is noted. Workup demonstrates no features of shunt, mass, or non-bacterial thrombotic endocarditis with normal left atrial size. Holter monitoring for 72 h did not detect the presence of atrial tachyarrhythmia. Her 10-year global risk of cardiovascular disease based on the Framingham 2008 calculator was 11.7%. 

### 1.2. Case 2

A 68-year-old man with polycythemia vera presents to the emergency department with chest pain. His current medications include low-dose aspirin, hydroxyurea, atorvastatin, and perindopril. He does not require regular phlebotomies for the management of polycythemia vera. Vital signs show a blood pressure of 93/72, HR 61, and oxygen saturation of 92% on 2 L by nasal prongs. Laboratory investigations reveal a hemoglobin of 142 g/L, platelets of 436 × 10^9^/L, WBC of 12.3 × 10^9^/L, and creatinine of 89 µmol/L. Troponin is elevated. ECG is diagnostic for an ST-elevation myocardial infarction. The patient is started on aspirin and intravenous unfractionated heparin. Cardiology is consulted.

## 2. Epidemiology of Arterial Thrombosis in Cancer

### 2.1. Overall Incidence

Solid tumors confer an approximately two-fold increase in the incidence of arterial thromboembolism (ATE) compared to age- and sex-matched population controls [1,2]. Data on ATE among patients with cancer can be estimated from both population-based cohort studies and control groups of thromboprophylaxis trials, in which ATE was captured as an efficacy endpoint [1,2,3,4].

Risk of cancer-associated ATE begins prior to an established diagnosis of cancer. A study using the Surveillance, Epidemiology, and End Results (SEER)–Medicare combined database in the United States has reported an incidence of myocardial infarction and ischemic stroke of 1.3% among patients within six months of a confirmed cancer diagnosis, compared to 0.6% among matched non-cancer controls (Table 1) [1]. Similarly, analysis of linked demographic, cancer, and prescription medication registries from the Danish administrative databases reported 1.5% and 0.6% rates of ATE (defined as myocardial infarction, ischemic stroke, and peripheral arterial disease) over the same timeframe [2]. A randomized, controlled trial (INCOGNITO trial; NCT05733416) is assessing the effectiveness of extensive cancer screening using positron emission tomography (PET) scan to diagnose occult cancers in patients with unexplained or cryptogenic stroke. Hence, clinicians should ensure that patients with ATE are up-to-date with age- and sex-specific cancer screening (i.e., colon, breast, cervix, and prostate). 

Risk of ATE continues to be elevated by approximately two-fold after a diagnosis of cancer, with a reported incidence of 4.7% among patients within the first six months of a cancer diagnosis, compared to 2.2% among matched controls in the SEER-Medicare cohort [1]. Meanwhile, 1.5% vs. 0.8% of patients with cancer and their matched controls developed an ATE within six months of diagnosis in the Danish administrative database study [2]. By 12 months of a new cancer diagnosis, cumulative incidences of ATE rose to 6.5% and 2.1% in the SEER-Medicare and Danish cohorts, respectively [1,2]. It is important to recognize that the SEER-Medicare database only included individuals ≥65 years and, therefore, comprised of an older group of patients compared to the Danish population-based study (median age 74 vs. 69 years).

Data from randomized, controlled trials that captured ATE as a co-efficacy endpoint support the cumulative incidences of ATE from observational cohorts (Table 1). The CASSINI trial, which randomized ambulatory patients with cancer at high risk of venous thromboembolism (VTE) to receive rivaroxaban 10 mg or placebo daily, reported a 1.7% cumulative incidence of ATE over six months of follow-up in the control arm [3]. Of note, all eligible individuals underwent duplex compression ultrasonography and were excluded if VTE was detected during this screening procedure [3]. Similarly, a 2% risk of ATE over 6 months was observed in the control group of the TARGET-TP trial, which tested the efficacy and safety of enoxaparin 40 mg daily to no anticoagulation among 200 ambulatory patients with lung and gastrointestinal cancers stratified to be at high-risk of cancer-associated VTE based on a model incorporating D-dimer and fibrinogen [5]. Taken together, the cumulative incidence of ATE falls in the range of 2% to 5% within six months of a new cancer diagnosis in the absence of pharmacologic thromboprophylaxis, which is approximately two-fold higher than the age- and sex-matched controls in the general population. 

Finally, the effect of cancer on ATE risk depends on the vascular bed involved. For example, data from the Danish population-based cohort study suggest the occurrence of peripheral artery disease (PAD) to be most strongly influenced by the presence of cancer, with hazard ratios (HRs) ranging from 13.8 to 38.8 among patients with lung and pancreatic cancers, respectively [2]. In contrast, HRs for myocardial infarction and ischemic stroke ranged from 0.6 to 1.8 across all primary cancer sites [2]. Nonetheless, the absolute risk increase in PAD from cancer remains less than 0.5% over the first year of cancer diagnosis, owing to its lower baseline incidence [2]. 

### 2.2. Risk Factors

In addition to deriving estimates of ATE incidence, cohort studies have determined patient-, cancer-, and treatment-related predictors of ATE [1,2]. In the Danish cancer cohort, patients with cancer diagnosed at 65–75 and >75 years had a 1.5- and 1.9-fold higher risk of ATE, respectively, compared to those aged <65 years at diagnosis, after accounting for the competing risk of all-cause mortality [2]. Additional demographic predictors of ATE in the same study included male sex (HR 1.15, 95% CI 1.08–1.22), traditional vascular risk factors, such as hypertension (HR 1.29, 95% CI 1.21–1.37) and diabetes (HR 1.20, 95% CI 1.10–1.29), and prior ATE (HR 2.96, 95% CI 2.77–3.17) [2]. Modification of subsequent ATE risk by a prior event was similarly reported in a study evaluating the subsequent risk of ischemic stroke among patients with cancer using data from Ontario, Canada [6]. Patients with a prior history of ischemic stroke had a 2.7-fold higher risk of subsequent ischemic stroke overall, which was especially increased among those with ischemic strokes within a year of cancer diagnosis (HR 3.68, 95% CI 3.22–4.22) [6]. 

Among cancer-related risk factors, the clinical stage of the underlying cancer was identified as a predictor of ATE. Metastatic disease was also associated with ATE onset, with HRs of 3.6 and 1.2 in the U.S. and Danish studies, respectively [1,2]. The impact of the primary site of cancer on ATE risk has not been fully elucidated to date and remains controversial: while primary cancer sites were not formally compared to each other, previous studies have reported hazard and incidence ratios of ATE attributable to specific cancer sites using non-cancer controls as a comparator. Pancreatic cancer was a strong predictor of ATE risk, with a HR of 4.8 (95% CI 3.93–5.83) in the Danish study and a cumulative incidence ratio of 2.5 in the U.S. cohort, compared to population controls [1,2]. Similarly, lung cancer conferred a 3.8- and 3.5-fold increase in ATE risk in these two studies, respectively [1,2]. While some primary cancer sites associated with increased VTE risk also increased the risk of ATE, other disease sites appeared to have a discordant impact on ATE and VTE risks. For example, while colorectal cancer is not considered a prothrombotic disease site for VTE, it was associated with 2.4- and 3.8-fold increases in ATE in the U.S. and Danish studies, respectively [1,2]. Whether this discrepancy relates to characteristics of the cancer type or to its treatment remains unknown. Furthermore, incidence of concomitant risk factors predisposing to ATEs may differ between sites of primary cancer. For example, incidence of atrial fibrillation (AF) was observed to be highest among patients with lung and gastrointestinal tumors in two population-based databases [7,8]. The impact of undetected or untreated AF on differences in ATE incidence between tumor sites, therefore, warrants further evaluation.

The relationship between cancer and ATE is further influenced by anti-cancer therapies. Several systemic therapies are associated with ATE, including conventional chemotherapy (e.g., alkylating agents, fluoropyrimidines, and anthracyclines), monoclonal antibodies (e.g., bevacizumab), and tyrosine kinase inhibitors [9,10,11,12,13]. While anti-cancer therapy is independently associated with increased ATE risk (HR 1.47, 95% CI 1.33–1.61) [2], three classes of agents warrant specific mention: anti-vascular endothelial growth factor (VEGF) inhibitors, BCR-ABL tyrosine kinase inhibitors (TKIs), and endocrine therapies. Treatment with anti-VEGF agents carries a high risk of hypertension as a class effect, while endothelial vascular damage from VEGF inhibition may synergistically increase ATE risk when used in combination with conventional chemotherapy [14]. In a systematic review and meta-analysis of randomized, controlled trials, anti-VEGF monoclonal antibodies and tyrosine kinase inhibitors were associated with a 1.6-fold higher risk of ATE compared to non-VEGF agents [15]. However, ATEs were captured as cardiovascular adverse events among the included trials, making precise effect size estimates challenging to obtain due to under-reporting of thrombotic complications in chemotherapeutic investigations [16]. Similarly, the advent of targeted TKIs has revolutionized the prognosis and treatment-related toxicities among patients with a wide array of malignancies. However, these advances have been accompanied by specific thrombotic complications, including ATE associated with BCR-ABL TKIs in the treatment of chronic myeloid leukemia and Philadelphia-positive acute lymphoblastic leukemia. Specifically, second- and third-generation BCR-ABL TKIs, nilotinib and ponatinib, are associated with a higher incidence of PAD among patients with chronic myeloid leukemia [17,18]. Recognizing this impact, the surveillance protocol advocated by the European Society of Cardiology Cardio-Oncology guideline emphasizes the use of the ankle-brachial index at baseline, 6 months, and every 6–12 months thereafter to assess for the presence of PAD among patients treated with these agents [19]. Finally, while endocrine therapies, such as aromatase inhibitors, have been associated with an increased risk of cardiovascular events in breast cancer compared to selective estrogen receptor modulators (SERM), a recent systematic review identified that these findings may be driven by the cardioprotective effects of SERM agents, such as tamoxifen [20]. Hence, patient-, cancer-, and treatment-related factors are all important contributors to the underlying incidence of ATE in patients with cancer.

Finally, a role of biomarkers in risk stratification of ATE is emerging. In a prospective multi-center Norwegian cohort of patients with pancreatic-cancer-associated VTEs, Larsen et al. reported a 36% cumulative incidence of breakthrough ATEs within the first six months of therapeutic apixaban treatment, primarily manifesting as ischemic strokes [21]. Leukocyte count was higher among patients with breakthrough ATEs compared to those without such events (10.7 vs. 6.8, *p* = 0.007). While thrombocytosis is a core feature of two non-BCR/ABL-mutated myeloproliferative neoplasms (MPNs, essential thrombocytosis and pre-fibrotic myelofibrosis), a screening study in Naestved, Denmark, involving over 19,000 community-dwelling individuals, identified a 3.1% prevalence of the JAK2 V167F mutation in the general population, of whom only 3% had an established MPN [22]. Individuals with the JAK2 V617F mutation (irrespective of MPN status) had a higher platelet count compared to their non-JAK2-mutated counterparts, and this was, in turn, associated with a higher risk of ischemic strokes (odds ratio 4.6, *p*-value = 0.03) [22]. Furthermore, a randomized, controlled trial among patients with high-risk essential thrombocytosis demonstrated a 20.4% absolute risk reduction in thromboembolism with a platelet target of <600 × 10^9^, compared to no cytoreduction, primarily driven by a reduction in arterial events [23]. This further highlights the role of thrombocytosis among patients with MPN. In terms of hemostatic markers, the TARGET-TP trial enrolled patients with lung and gastrointestinal malignancies at high risk of thromboembolism based on fibrinogen and D-dimer levels, and a numerically higher rate of ATE was observed among high- vs. low-risk patients who did not receive enoxaparin thromboprophylaxis (2.0% vs. 0.8%, adjusted hazard ratio 1.52, 95% confidence interval 0.1 to 24.3) [5]. 

## 3. Pathophysiology of Arterial Thrombosis in Cancer

The pathophysiology of cancer and ATE is complex. While some mechanisms appear to be cancer-specific, others are common across several types of cancers, involving a broader activation of the immune and coagulation systems [24]. The interplay between cancer and ATE is further complicated by the observation that several risk factors associated with the development of arterial thrombosis have also been associated with a higher risk of cancer [25].

Myeloproliferative neoplasms (MPN) are cancers of myeloid blood cells. They are divided by the presence of the BCR-ABL mutation into BCR-ABL-positive and BCR-ABL-negative cancers [26]. BCR-ABL-negative MPN (including polycythemia vera, essential thrombocythemia, and primary myelofibrosis) are highly associated with thrombosis, which represents a main cause of mortality and morbidity in this chronic cancer population [27]. In BCR-ABL-negative MPN patients, multiple cancer-related changes intertwine to create this pro-thrombotic environment, starting with the cancer’s driver mutation itself [28,29,30]. These cancers offer the opportunity of reviewing several cancer-specific pathways associated with increased thrombotic risk. The JAK2 mutation, a gain-of-function mutation leading to the proliferation of myeloid cells, is the most common driver mutation in BCR-ABL-negative MPN [26]. This specific mutation has been found in both hematopoietic and endothelial cells of MPN patients. It has been hypothesized that this finding is due to the presence of a common hematopoietic and endothelial cell progenitor cell harboring the JAK2 mutation [31,32,33]. Hyper-viscosity caused by erythrocytosis is also often cited as the cause of thrombosis in JAK2-positive MPN. The seminal CYTO-PV study has shown that a lower hematocrit target of less than 45% (achieved through phlebotomy and/or cytoreduction) is associated with a lower risk of cardiovascular death and major thrombosis compared to a higher hematocrit target [34]. Beyond simple quantitative changes, though, several qualitative changes to the endothelium and blood cells associated with the JAK2 mutation also heighten the thrombotic risk (i.e., ATE and VTE). This mutation leads to a pro-adhesive environment, enhancing the interactions between the endothelium and blood cells, which in turns promotes thrombus formation. The JAK2-positive endothelial cells are associated with a higher exposure of P-selectin due to Weibel–Palade body degranulation, which translates into increased interactions between endothelium, leucocytes, and platelets [35]. The presence of P-selectin also upregulates tissue factor expression on monocytes and the concentration of tissue factor at sites of developing thrombus [36]. Furthermore, the JAK2 mutation causes the phosphorylation of the laminin a5 chain and the Lutheran/basal cell adhesion molecule (Lu/BCAM) on erythrocytes. This, in turn, increases the interactions between erythrocytes and endothelial cells. Heightened erythrocyte and platelet interactions have also been described, with greater expression of phosphatidylserine stimulating thrombin generation [28,37]. Furthermore, inflammation appears to play a role in the thrombotic risk of MPN patients. This thrombo-inflammatory state comes from the interplay between abnormal blood cells, dysfunctional endothelium, and activated coagulation cascade. It has been suggested that inflammation may promote the selective clonal evolution of the JAK2 mutation and other MPN driver mutation-bearing hematopoietic progenitors, suppressing the wild-type population. Conversely, JAK2 mutation signaling also drives inflammatory pathways, leading to a self-sustaining pathological state [30]. 

Broader mechanisms associated with cancer development and progression also seem to play a role in increased ATE. Recent studies have suggested that neutrophil extracellular traps (NETs) are associated with thrombosis, as well as cancer development [38,39]. Of particular note, platelets also seem to play an important role in cancer-associated ATE. Histological comparison of thrombus in stroke patients with active cancer showed a higher fraction of platelets and lower fraction of erythrocytes, when compared with patients with inactive cancer or without any history of cancer, perhaps highlighting the need to tailor antithrombotic therapies in this population [40]. Several cancer lines have been shown in vitro to secrete platelet activators, such as matrix metalloproteinases, thrombin, ADP, thromboxane A2, and tumor-derived vascular endothelial growth factors [41].

## 4. Prevention of Arterial Thromboembolic Events

### 4.1. Primary ATE Prevention in Cancer Patients without Established Indication for Anticoagulation

The procoagulant nature of cancer and its treatment has led to numerous interventional trials aiming at evaluating the efficacy and safety of different anticoagulant regimens for the primary prevention of cancer-associated VTE. While pharmacologic thromboprophylaxis in ambulatory patients with cancer initiating systemic therapy using thromboprophylactic dosing of anticoagulants (low-molecular-weight heparin (LMWH) or direct oral anticoagulants (DOAC), i.e., apixaban and rivaroxaban) has been demonstrated to be efficacious, safe, and cost-effective for the prevention of cancer-associated VTE [42,43,44], clinical equipoise persists on whether anticoagulants prevent ATE in this patient population. In a systematic review and meta-analysis of 11 randomized, controlled trials with 10,248 patients, thromboprophylactic use of anticoagulation was not associated with a decrease in ATE risk compared to no anticoagulant use (relative risk (RR) 0.73, 95% CI 0.50–1.04, *p* = 0.08) [45]. While the upper bound of the 95% confidence interval in the study included the possibility of a 0.67% absolute risk reduction in ATE [45], this falls below minimal clinically important differences used in contemporary ATE trials involving oral anticoagulants [46,47]. 

While thromboprophylaxis may not be efficacious for ATE prevention in ambulatory cancer patients overall, there may be subgroups with additional ATE-related risk factors in whom primary thromboprophylaxis may prevent such events. For example, subgroup analyses of a systematic review and meta-analysis reported that thromboprophylaxis may be associated with a reduced risk of ATE among patients with pancreatic cancer without a detectable increase in the risk of major bleeding [48]. Hence, derivation and validation of clinical prediction tools for cancer-associated ATE to identify such high-risk patients is, therefore, a research priority, in order to evaluate novel prevention strategies aimed at reducing the overall burden of cancer-associated ATE with adequately powered interventional study designs. 

Finally, anti-platelets have a role for primary ATE prevention in certain malignancies, especially MPNs. For example, the European Collaboration on Low-dose Aspirin in Polycythemia Vera (ECLAP) trial showed a numerical decrease in the composite endpoint of nonfatal myocardial infarction, nonfatal stroke, or death from cardiovascular causes with use of aspirin 100 mg daily compared to placebo among patients with polycythemia vera (4.9% vs. 2.0% over three years, *p* = 0.09). The finding was statistically significant when VTE was added as part of the efficacy endpoint (7.9% vs. 3.2%, *p* = 0.03) [49]. 

### 4.2. Primary ATE Prevention Patients with Cancer and AF

While robust international guidelines exist on risk stratification and prevention of stroke and systemic embolic events among patients with AF in the general population [50,51,52], risk stratification among patients with cancer is challenging due to the increased risk of both ATE and bleeding complications. Emerging data suggest that cancer is an independent risk factor among patients with AF or atrial flutter. For example, a recent study included patients with concomitant cancer and AF, and focused on patients with CHA_2_DS_2_-VASc scores of 0, 1, and 2, which correspond to thresholds considered to be at low or intermediate risk of ATE in the general population [53]. Using data from a population-based cohort of 29,298 individuals, the investigators reported a 1.4% cumulative incidence of ATE within 12 months from cancer diagnosis among non-anticoagulated patients deemed at low risk of stroke or systemic embolism (defined as CHA_2_DS_2_-VASc = 0 among men and CHA_2_DS_2_-VASc = 1 among women), compared to 0.5% in non-cancer controls [53]. The risk difference in the intermediate risk category (defined as CHA_2_DS_2_-VASc = 1 among men and CHA_2_DS_2_-VASc = 2 among women) was more striking, with ATEs occurring in 2.1% vs. 0.4% among cancer and non-cancer patients in the absence of anticoagulation, respectively [53]. Along with results from the Spanish CardioCHUVI-AF registry and a Danish national cohort analysis [54,55], increased ATE risk in patients with concomitant cancer and AF has led to calls to action for the addition of cancer to the CHA_2_DS_2_-VASc score to improve risk stratification [56]. On the other hand, anticoagulant-associated bleeding among patients with cancer and AF is higher due to multiple etiology, including prolonged thrombocytopenia from bone marrow infiltration and myelosuppressive agents, off-target effects of conventional and targeted treatments, as well as frequent need for procedural interventions. In the ENGAGE AF TIMI 48 trial, those with active cancer experienced a 2.5-fold higher incidence of anticoagulant-associated major bleeds compared to their non-cancer counterparts (7.4 events vs. 2.5 events per 100 person-years, HR 2.5, 95% CI 2.1–2.9) [57]. 

Despite emerging data on risk stratification for stroke and systemic embolism, comparative data of anticoagulant agents in patients with concomitant cancer and AF are scant. Despite the advent of DOACs that significantly reduced the risk of major bleeding compared to vitamin K antagonists (VKA) in phase three randomized, controlled trials of patients with non-valvular AF [58], patients undergoing active cancer treatment were under-represented in these pivotal studies (Table 2). Patients with active cancer comprised less than 1% of participants in both ROCKET-AF and ARISTOTLE trials, which evaluated the efficacy and safety of rivaroxaban and apixaban, respectively [59,60]. Meanwhile, a post hoc analysis of the ENGAGE-AF TIMI 48 trial (n = 21,105) compared ATE and bleeding outcomes in patients who developed new or recurrent cancers to those who remained cancer-free during the study follow-up [57]. Of 21,105 randomized patients, 5.5% (1153) developed an active cancer during the median trial follow-up of 2.8 years, with equal distribution between the edoxaban and VKA arms [57]. Gastrointestinal tumors were the most diagnosed site of primary cancer, and the time in therapeutic range of patients randomized to VKA was 68% [57]. Interestingly, patients randomized to edoxaban experienced fewer ischemic events (defined as composite of stroke, systemic embolism, and myocardial infarction) compared to those treated with VKA, which contrasted with the overall trial findings of non-inferiority between the two arms [57]. Treatment-emergent bleeding events were similar between the two arms, and the incidence of International Society on Thrombosis and Hemostasis (ISTH)-defined major bleeding was 7.9 vs. 8.2 events per 100 person-years in the edoxaban and warfarin arms, respectively (HR 0.98, 95% CI 0.69–1.40) [57]. This post hoc analysis, therefore, suggests that edoxaban may confer higher efficacy for ATE prevention in AF compared to VKA, at the expense of the improved safety profile observed in non-cancer patients. It is relevant to acknowledge that major bleeding definitions established as the safety endpoint in clinical evaluations of antithrombotic therapies are composite endpoints [61], with varying degrees of prognostic significance across component criteria [62]. Some criteria, such as a hemoglobin decrement of 20 g/L or more, may occur more frequently among patients with cancer due to competing, non-hemorrhagic etiologies (e.g., myelosuppressive chemotherapy), thereby inflating their true bleeding risk on antithrombotic therapy.

While guidelines from the ISTH and European Society of Cardiology (ESC) suggest DOACs as first-line treatment among patients with cancer and new diagnosis of AF who meet the indication for anticoagulation [19,63], selection of the optimal anticoagulant agent in patients who are unsuitable for DOAC treatment (e.g., moderate to severe thrombocytopenia, drug–drug interactions, etc.) remains challenging. While LMWHs are suggested in this scenario by the ESC guideline [19], this class of agents has primarily been evaluated in the treatment of VTE in cancer without high-quality evidence comparing LMWHs to oral anticoagulants in patients with AF and cancer. The comparative effectiveness and safety of patients with cancer and AF treated with long-term LMWH are, therefore, unknown. While the three-month cumulative incidence of thrombocytopenia (defined as platelet count < 100 × 10^9^) in patients receiving chemotherapy is estimated at 12.8% in solid tumors and 28.2% in hematologic malignancies [64], patients with a platelet count < 90–100 × 10^9^/L were excluded from pivotal phase III trials involving DOACs in AF [65,66,67,68]. ATE and bleeding outcomes of anticoagulated patients with AF and moderate thrombocytopenia were reported in a post hoc analysis of the AFIRE trial, which compared dual and single antithrombotic therapy among patients with concurrent AF and coronary artery disease, including non-cancer patients with mild and moderate thrombocytopenia ≥ 50,000/mm^3^ [69,70]. Compared to patients receiving rivaroxaban monotherapy with a normal platelet count, the presence of thrombocytopenia (defined by platelet count < 100,000/mm^3^) was associated with a numerically increased risk of major bleeding over three years of follow-up (5.7% vs. 3.2%, HR 2.0, 95% CI 0.48–8.34) [70]. This finding is also supported by data from a single-center retrospective cohort study reporting that patients with a baseline platelet count < 75,000/mm^3^ on oral anticoagulation experienced a 2.9-fold increase in ISTH-defined major bleeding complications compared to those with normal platelet counts [71]. However, incidence of ATE was numerically higher among thrombocytopenic patients in both studies compared to their non-thrombocytopenia counterparts (8.6% vs. 5.4% in the AFIRE trial; 3.6% vs. 1.5% in a single-center cohort) [70,71]. The net clinical benefit of anticoagulation among patients with cancer and thrombocytopenia, therefore, warrants further evaluation. Acknowledging existing data limitations, the European Hematology Association (EHA) guideline provides recommendations for therapeutic anticoagulation at approved dosing among patients with non-valvular AF with stable moderate grade 1 (platelets count 75,000–100,000/mm^3^) or grade 2 (platelets count 50,000–75,000/mm^3^) thrombocytopenia after reviewing risk factors for ATE and bleeding complications [72]. 

## 5. Management of Arterial Thrombosis in Cancer Patients

The management of ATE in patients with cancer represents a particularly difficult clinical challenge. Cancer patients are at a risk of higher bleeding than the general population, often suffer from thrombocytopenia, and are exposed to potentially significant drug–drug interactions [73,74,75,76]. Consequently, ATE may be undertreated in patients with cancer, who remain at higher risk of mortality and recurrent ATE. These considerations affect both the risk–benefit assessment of antithrombotic therapy and the choice of antithrombotic therapy. Unfortunately, there is limited data to guide clinical conduct in the management of ATE in cancer patients.

### 5.1. Acute Coronary Syndrome

The diagnosis of acute coronary syndrome (ACS) in patients with cancer follows the same diagnostic algorithm as in non-cancer patients [19]. It is necessary, however, to maintain an increased suspicion for ACS in cancer patients presenting with various complaints. In a retrospective review of ACS management in patients with cancer, dyspnea, instead of chest pain, was the most common presenting symptom of ACS [77]. Furthermore, it may be necessary to distinguish ACS due to plaque rupture from other cancer-specific pathologies with similar clinical presentations, such as cancer-therapy-related cardiac dysfunction, vasospasms, or myocarditis [78,79,80].

The ESC, in collaboration with the EHA, the European Society for Therapeutic Radiology and Oncology, and the International Cardio-Oncology Society, has recently proposed guidelines on the topic of cardio-oncology [19]. For the management of ACS, the clinical practice guidelines are informed by observational data, and most of the recommendations are based on expert consensus. These guidelines propose that in patients with an expected survival of six months or more, invasive approaches for the management of ACS are recommended. In patients with an expected survival of less than six months or at very high bleeding risk, conservative, non-invasive management strategies should be considered. Anti-neoplastic treatment interruption is recommended if the anti-neoplastic drug is a suspected causative factor. However, this is a 1C recommendation, meaning that although it is a strong recommendation, it represents expert consensus in the context of very limited data. At minimum, a thorough review of all anti-neoplastic medications needs to be undertaken to assess for medications with known cardiac toxicities or leading to severe thrombocytopenia (in preparation for the use of antithrombotic therapies). Since cancer patients are at a higher risk of bleeding, the shortest course of dual anti-platelet therapy is recommended. Overall, it is emphasized that a multidisciplinary approach, regrouping cardiology, oncology, and hematology specialists, is necessary.

### 5.2. Ischemic Stroke

The type of treatment proposed for the management of ischemic stroke in cancer patients is based on the etiology of the stroke itself. Similarly to patients without cancer, a thorough and systemic assessment is required to identify the stroke etiology. Where applicable, traditional cardiovascular risk factors should be optimized [81,82]. In cancer patients, however, specific stroke etiologies deserve special attention.

A thorough cardiac structural assessment is usually indicated for the management of non-lacunar ischemic stroke. In cancer patients, a transesophageal echocardiogram (TEE) is often warranted (when a transthoracic echocardiogram is negative) since TEE may provide additional information, which will ultimately influence treatment decisions [83]. Patients with cancer are at high risk of VTE [84]. In the context of a Patent Foramen Ovale (PFO), this may lead to a paradoxical stroke. Assessment for a PFO, a common cardiac malformation in the general population [85], is therefore a usual step in the determination of stroke etiology, but is of particular importance in patients with cancer due to their known hypercoagulability. Of note, when investigating specifically for a PFO, transcranial doppler, compared to the more invasive TEE, has been shown to have a 96.1% (95% CI 93.0–97.8) sensitivity and a 92.4% (95% CI 85.5–96.1) specificity for the detection of a right-to-left shunt, usually associated with a PFO [83]. A focused history and physical examination for deep vein thrombosis and pulmonary embolism at the time of stroke presentation is also indicated. In patients with cancer, it may be appropriate to perform screening compression ultrasonography of the lower extremities to further assess for the presence of deep vein thrombosis, considering the increased risk for VTE [86]. PFO management following a stroke is determined by the probability that the PFO played a causal role in the stroke presentation. In patients where the PFO is thought to be associated with the stroke, discussions with neurologists and cardiologists are needed to decide on the most appropriate management option [81]. However, in patients with cancer, it may be appropriate to initiate anticoagulation therapy (even without a concomitant VTE) to prevent recurrent stroke in patients with a PFO and high-risk anatomical features since it increases the likelihood of a relationship with the stroke [87] and cancer is a persistent risk factor for VTE. This decision must be individualized based on patient preferences, bleeding risk, and the consideration of other management options (e.g., PFO closure).

Nonbacterial thrombotic endocarditis (NBTE) may also be identified with appropriate cardiac structural assessment as a cause of stroke in patients with cancer. Little is known about the pathogenesis of NBTE, but it is associated with hypercoagulable states, such as cancer, where hypercoagulability leads to the formation of non-infectious valvular vegetations [88]. NBTE management to prevent stroke recurrence first requires management of the underlying neoplastic process, which is thought to be the driving force of the hypercoagulable state. Expert consensus also advocates for the use of anticoagulation to further decrease the risk of recurrent stroke, with a preference for LMWH as the anticoagulant therapy of choice [89,90].

Despite standardized evaluation, the etiology of the stroke often remains unexplained [91,92]. Cryptogenic strokes are common in both the cancer and non-cancer populations [93]. A subset of cryptogenic strokes that are non-lacunar are referred to as embolic strokes of undetermined source (ESUS) [94]. In the cancer population, markers of a hypercoagulable state, such an elevation in D-dimer, are often noted [93,95]. It has been proposed that hypercoagulability from cancer processes can lead to ESUS through the formation of in situ thrombi due to cerebral intravascular coagulation [86]. There is considerable equipoise regarding the management of ESUS. Recently, two randomized, controlled trials, NAVIGATE-ESUS [96] and RE-SPECT ESUS [97], have compared DOACs to aspirin for the management of ESUS. In these trials, the use of DOACs was not associated with improved thrombotic outcomes but led to increased bleeding complications. When looking specifically in the subpopulation of patients with cancer in the NAVIGATE-ESUS trial (7.5% of the trial population), the same observations regarding the lack of a significant difference in stroke outcome between the two groups were observed, along with increased bleeding in receiving rivaroxaban. It is important to note, however, that the number of patients with active cancer was not reported [98]. As such, in the case of ESUS in cancer patients, current evidence, including limited data from randomized, controlled trials and observational studies [86], does not support the use of anticoagulation over an anti-platelet agent as the standard treatment. Due to the lack of high-quality data in this field, however, an individualized approach, incorporating the patient’s perceived bleeding risk and preferences, may be used to justify anticoagulation therapy, although it should not be adopted as the default treatment approach.

### 5.3. Peripheral Artery Disease 

There is limited evidence to guide the management of peripheral artery disease (PAD) in cancer patients. It is, however, important to note that certain therapies, such as the BCR-ABL tyrosine kinase inhibitors, nilotinib and ponatinib, have been associated with an increased risk of developing PAD [99,100]. As with other arterial thromboembolic complications in this patient population, a multidisciplinary approach to guide management is recommended. This approach is especially important regarding the decision on the cessation or interruption of anti-neoplastic medications, which are thought to be associated with the development of PAD [19].

## 6. Approach for the Management of Arterial Thrombosis in Cancer Patients

We propose a generalized approach for the management of ATE in patients with cancer. We highlight the need for a multidisciplinary team to guide management of these complex patients, where appropriate, standard-of-care treatment options for ATE should be considered. In certain circumstances, however, a more tailored approach is needed, with concrete examples provided below.

1. Review of non-cancer etiology for ATE

Considering the shared risk factors for both cancer and ATE (e.g., age as a risk factor for malignancy, AF, and atherosclerotic cardiovascular disease), it is crucial to review the evaluation to date prior to attributing cancer and its treatment as the major risk factor for ATE. Specifically, imaging characteristics warrant review with organ-specific radiology to determine the potential underlying vascular etiology [101].

2. Assessment of goals of care and life expectancy

Patients with a reduced life expectancy are often excluded from clinical trials, making the risk–benefit assessment for various therapies more challenging. In many cases, medications that lead to a small thrombotic risk reduction over a prolonged period of time are unlikely to be of overall benefit. Similarly, the risk–benefit profile of invasive procedures with the potential for immediate complications or leading to an intensified subsequent use of antithrombotic therapy (e.g., percutaneous cardiac intervention with drug-eluting stents, etc.) must be carefully weighed. This assessment regarding overall cancer prognosis should include the patient’s oncology specialists to fully appreciate the larger issues at play.

3. Review of medications

A thorough review of medications is necessary to assess for a potential cause of the ATE, to evaluate for potential drug–drug interactions with the standard therapy, and to anticipate future drug-related complications (e.g., thrombocytopenia, etc.), which may increase a patient’s bleeding risk. Where the anti-neoplastic treatment is associated with an increased risk of ATE, interruption of this medication may be considered in specific situations, although this must be performed in discussion with the patient’s treating oncologist and with the acknowledgement of the potential detrimental effect of this interruption on the overall cancer journey.

4. Assessment of bleeding risk

Bleeding risks must be sought out before deciding on an antithrombotic agent, thrombolysis, or invasive procedure. In addition to the general risk factors for bleeding (e.g., prior clinically significant bleeding, anemia, advanced age, renal dysfunction, liver dysfunction, etc.), cancer-specific risk factors associated with the tumor itself (whether described as hemorrhagic or invasive of vasculature), cancer-medicated coagulopathy and thrombocytopenia, and drug-related derangements in the coagulation process must also be considered.

5. Review standard-of-care management options, with adjustments as needed

For the management of ATE, anti-platelets remain the standard of care, as in the general population. It is incumbent to involve the organ-specific specialist (such as neurologist, cardiologist, or vascular surgeon) prior to treatment planning. Special considerations in the cancer population would include cancer- or anti-neoplastic-specific mimickers of ACS, as well as more thorough investigations in specific stroke etiologies (paradoxical stroke in the context of PFO, NBTE, and ESUS).

## 7. Case Resolution

Case 1. Considering the multi-territorial cerebral infarct, concerns for an embolic event were raised. After review of the diagnostic workup that did not reveal an alternate cause requiring therapeutic anticoagulation, ASA 162 mg load followed by 81 mg daily was initiated considering the concern for risk of early hemorrhagic transformation with therapeutic anticoagulation after consensus discussion between neurology, hematology, and medical oncology. The patient experienced hemoptysis secondary to her cancer, which resolved after treatment with radiation therapy. The patient was resumed on ASA 81 mg with no recurrent ATEs or bleeding events during follow-up.

Case 2. In the context of polycythemia vera, which is a chronic cancer, and in the absence of strong bleeding risk factors, the patient was assessed as a good candidate for standard-of-care treatment of an ST-elevation myocardial infarction. As such, a percutaneous coronary intervention took place, leading to the placement of two drug-eluting stents. Post-procedure, dual anti-platelet therapy with aspirin and clopidogrel was initiated. Optimization of the patient’s cardiovascular risk factors was undertaken through behavioral modifications and pharmacological therapies. Close monitoring of the patient’s hematocrit level was also arranged by his hematologist.

## 8. Conclusions

Cancer patients are at high risk of arterial thrombotic complications. There is limited data to guide the prevention and management of arterial thrombosis in this patient population. Additional research to better understand the pathophysiology of arterial thrombosis in cancer, as well as to guide the prevention and management of arterial thromboembolic complications, is eagerly awaited.

## Figures and Tables

**Table 1:** Risk of arterial thromboembolism (ATE) among patients with cancer not on routine pharmacologic thromboprophylaxis.

Study	ATE Outcome Measure	Subgroup	6 Months before Cancer	Within 3 Months of Cancer	Within 6 Months of Cancer	Within 12 Months of Cancer
SEER + Medicare database (≥65 years) [1]	Myocardial infarction, ischemic stroke (non-adjudicated ICD code)	Cancer (n = 279,719)	1.3%	3.4%	4.7%	6.5%
		Matched control (279,719)	0.6%	1.1%	2.2%	4.2%
Danish cancer + national registry [2]	Myocardial infarction, ischemic stroke, peripheral arterial occlusion (non-adjudicated ICD code)	Cancer (n = 458,462)	1.5%	N/A	1.5%	2.1%
		Matched control (n = 1,375,386)	0.6%		0.8%	1.5%
CASSINI trial [3]	Myocardial infarction, stroke, peripheral arterial disease (adjudicated secondary efficacy endpoint)	Cancer (n = 421)	N/A		1.7%	N/A
TARGET-TP trial [5]	Myocardial infarction (adjudicated co-efficacy endpoint)	Cancer (n = 100)	N/A		2.0%	N/A
INPACT trial [4]	Myocardial infarction, ischemic stroke, systemic embolism (adjudicated secondary efficacy endpoint)	Cancer (n = 244)	N/A			3.9%

N/A: not available.

**Table 2:** Characteristics of patients with active cancer enrolled in phase three trials of direct oral anticoagulants in atrial fibrillation.

Study	Age	Female (%)	Primary Site of Malignancy	Mean CHADS_2_	How Cancer Was Defined	Stroke and Systemic Embolism	Major Bleeding
ROCKET-AF (n = 48/14,264) [60]	77 (IQR 72–81)	52	Prostate: 29% Colorectal: 16% Breast: 15% Genitourinary: 12% Lung: 3%	3.5 (48% prior stroke/SE)	640 patients with history of cancer; 48 actively treated	R: 1/48 W: 1/48	R: 1/50 * W: 2/50
ARISTOTLE (n = 157/18,183) [59]	74 (IQR 68–80)	20	Prostate: 42% Lung: 16% Breast: 11% Colorectal: 9% Bladder: 9%	2.2 (19% prior stroke)	Actively treated cancer (1079 remote)	A: 0/76 W: 5/81	A: 1/76 W: 5/81
ENGAGE-AF TIMI 48 (n = 1153/21,105) [57]	75 (IQR 68–79)	31	Gastrointestinal: 21% Prostate: 14% Lung: 11% Bladder: 8% Breast: 7%	2.8 (25% prior stroke)	New or recurrent cancer during follow-up	E: 1.4 per 100 patient-years W: 2.4 per 100 patient-years	E: 7.9 per 100 person-years W: 8.2 per 100 person-years

* On-treatment population; R, rivaroxaban; W, warfarin; A, apixaban; E, edoxaban.
